# Defective Uteroplacental Vascular Remodeling in Preeclampsia: Key Molecular Factors Leading to Long Term Cardiovascular Disease

**DOI:** 10.3390/ijms222011202

**Published:** 2021-10-18

**Authors:** Kirim Hong, Soo Hyun Kim, Dong Hyun Cha, Hee Jin Park

**Affiliations:** Department of Obstetrics and Gynecology, CHA Gangnam Medical Center, CHA University, Seoul 06125, Korea; rachelkh@chamc.co.kr (K.H.); soohyunkim@chamc.co.kr (S.H.K.); chadh001@chamc.co.kr (D.H.C.)

**Keywords:** preeclampsia, spiral artery remodeling, cardiovascular disease

## Abstract

Preeclampsia is a complex hypertensive disorder in pregnancy which can be lethal and is responsible for more than 70,000 maternal deaths worldwide every year. Besides the higher risk of unfavorable obstetric outcomes in women with preeclampsia, another crucial aspect that needs to be considered is the association between preeclampsia and the postpartum cardiovascular health of the mother. Currently, preeclampsia is classified as one of the major risk factors of cardiovascular disease (CVD) in women, which doubles the risk of venous thromboembolic events, stroke, and ischemic heart disease. In order to comprehend the pathophysiology behind the linkage between preeclampsia and the development of postpartum CVD, a thorough understanding of the abnormal uteroplacental vascular remodeling in preeclampsia is essential. Therefore, this review aims to summarize the current knowledge of the defective process of spiral artery remodeling in preeclampsia and how the resulting placental damage leads to excessive angiogenic imbalance and systemic inflammation in long term CVD. Key molecular factors in the pathway—including novel findings of microRNAs—will be discussed with suggestions of future management strategies of preventing CVD in women with a history of preeclampsia.

## 1. Introduction

Preeclampsia is a subtype of hypertensive disorders of pregnancy (HDP), defined as hypertension (systolic blood pressure ≥ 140 mmHg and/or diastolic blood pressure ≥ 90 mmHg) newly developed at or after 20 weeks of pregnancy with at least one of following conditions: proteinuria (≥1 + dipstick; ≥30 mg/mmol protein:creatinine ratio; or ≥300 mg/24 h), maternal organ dysfunction (hepatic, renal, hematological, or neurological conditions), or uteroplacental dysfunction (such as abnormal umbilical artery Doppler wave form analysis, fetal growth restriction, or stillbirth) [1]. The incidence of preeclampsia is reported to be 5–7% of all pregnancies, which can cause fatal conditions in the mother and the newborn [2].

Besides the higher risk of unfavorable obstetric outcomes in women with preeclampsia, another crucial aspect that needs to be considered is the association between preeclampsia and the postpartum cardiovascular health of the mother. According to the American Heart Association, preeclampsia is classified as one of major risk factors of cardiovascular disease (CVD) in women [3]. In fact, according to a large scaled meta-analysis, a history of preeclampsia doubles the risk for subsequent venous thromboembolic events, stroke, and ischemic heart disease over five to 15 years postpartum in women [4]. While some of the maternal systemic damage by preeclampsia returns to normal after delivery, it has become apparent that the vascular dysfunction by several molecular factors persists beyond the acute disease in pregnancy. Therefore, in order to comprehend the pathophysiology behind the linkage between preeclampsia and postpartum CVD, a thorough understanding of the abnormal uteroplacental vascular remodeling is essential.

## 2. Uteroplacental Vascular Development in Normal Pregnancy

The development of placental vasculature begins from the beginning of pregnancy as the blastocyst implants into the decidua. The cytotrophoblasts which originate from the extra-embryonic membranes of the fertilized ovum mediate this process by differentiating into endothelial cells as they invade into the uterine wall to form primary capillaries of placental vasculature [5]. As the implanted embryo develops, trophoblast cells continue to branch into the inner third of the myometrium and reach the maternal spiral arteries at the intervillous space where maternal-placental circulation occurs. Uterine spiral arteries are nonbranching end arteries of uterine arteries which penetrate the inner part of the myometrium and the endometrium with a corkscrew shape [6]. During pregnancy, the spiral arteries are responsible for providing adequate perfusion of uteroplacental blood flow. Therefore, the spiral arteries are physiologically modified in order to change from high-resistance vessels to dilated low-resistance vessels with a thin wall [7]. The process of so-called “spiral artery remodeling” has been suggested to have five stages according to Pijenborg et al. [8]. Stage 1 involves the swelling of individual smooth muscle cell in the uterine spiral artery along with endothelial vacuolation. Stage 2 begins with interstitial trophoblasts invading the perivascular tissues and disorganizing the vascular smooth muscle layer. It is followed by the appearance of endovascular trophoblasts (stage 3) and the trophoblast becomes embedded into the vessel wall, becoming intramural trophoblasts in stage 4. In stage 5, the re-endothelialization with newly built endothelium and the thickening of subintima containing myofibroblasts occur. During the process, several regulatory factors are involved; the high oxygen concentration in the spiral artery initiates the endovascular trophoblast invasion and activation of maternal decidual natural killer cells and platelets enhance their invasion [9]. Therefore, eventually the spiral arteries are physiologically altered to exhibit low vascular resistance and enhanced vasodilation, and this is specifically designed to provide sufficient uteroplacental circulation, which is critical for a successful pregnancy.

## 3. Defective Uteroplacental Vascular Remodeling in Preeclampsia

The association with failed spiral artery remodeling in development of preeclampsia was first brought up in 1972 by Brosens et al. [7]. Subsequent studies have revealed that due to a failure in the process of endovascular trophoblast invasions, spiral arteries fail to go through the physiological alteration process which results in relatively narrow, thick-walled and tortuous vessels in preeclampsia. Moreover, unlike in a normal pregnancy in which the transformation of the spiral artery extends from the decidual segment to one-third of the myometrial segment, in preeclampsia trophoblasts fail to invade into the myometrial segment of spiral arteries [10]. As a consequence, deep placentation fails and the blood flow to the placenta is restricted leading to inadequate uteroplacental perfusion. This phenomenon is found in various adverse pregnancy outcomes along with preeclampsia, such as fetal growth restriction, placental abruption, preterm labor, preterm premature rupture of membranes, and intrauterine fetal death [11,12,13,14]. 

## 4. Two Step Model of Preeclampsia

In order to understand how the defective spiral artery remodeling leads to preeclampsia, a thorough comprehension of the two step model is crucial. Preeclampsia manifests differently depending on the onset of the disease where the early onset form (develops before 34 weeks of gestation) is highly related to complications associated with placental dysfunction, such as a higher rate of fetal growth restriction, reduced placental volume, and low birth weight [15], while the late onset form of preeclampsia (develops at or after 34 weeks of gestation) is considered as a maternal syndrome without poor placentation, which rarely results in fetal growth restriction or low birth weight [16]. In fact, incomplete spiral artery remodeling is mostly seen in the early onset type of preeclampsia which serves as the ‘extrinsic’ cause of poor placentation. On the other hand, the late onset preeclampsia exhibits normal physiologically transformed spiral arteries with ‘intrinsic’ cause of placental malperfusion, caused by overcrowding of terminal villi as placental growth exceeds its functional limit. Overall, the abnormal intervillous blood flow results in ischemic-reperfusion injury and generates synciotrophoblast hypoxia stress [17]. Hence, although the pathways differ in their origin they both eventually lead to the maternal syndrome of preeclampsia resulting in high blood pressure and organ dysfunctions. 

**Figure 1:**
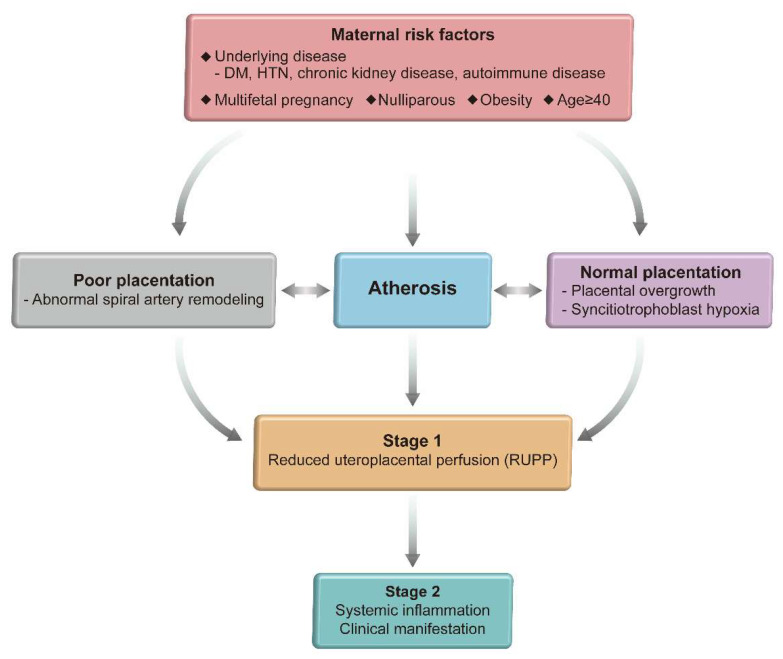
Two step model of preeclampsia.

Therefore, the updated two-step model describes how the different two types of pathways lead to the development of clinically recognized preeclampsia [16]. As described in Figure 1, stage 1 represents the placental dysfunctional stage with syncytiotrophoblast stress which results either from extrinsic or intrinsic cause as described earlier. In order for the disease to manifest as clinical syndrome of preeclampsia (stage 2), inflammatory factors produced by syncytiotrophoblast stress in stage 1 activates the maternal endothelium resulting in generalized vascular inflammation and endothelial dysfunction leading to maternal clinical signs of hypertension and organ failure. 

## 5. Atherosis

**Figure 2:**
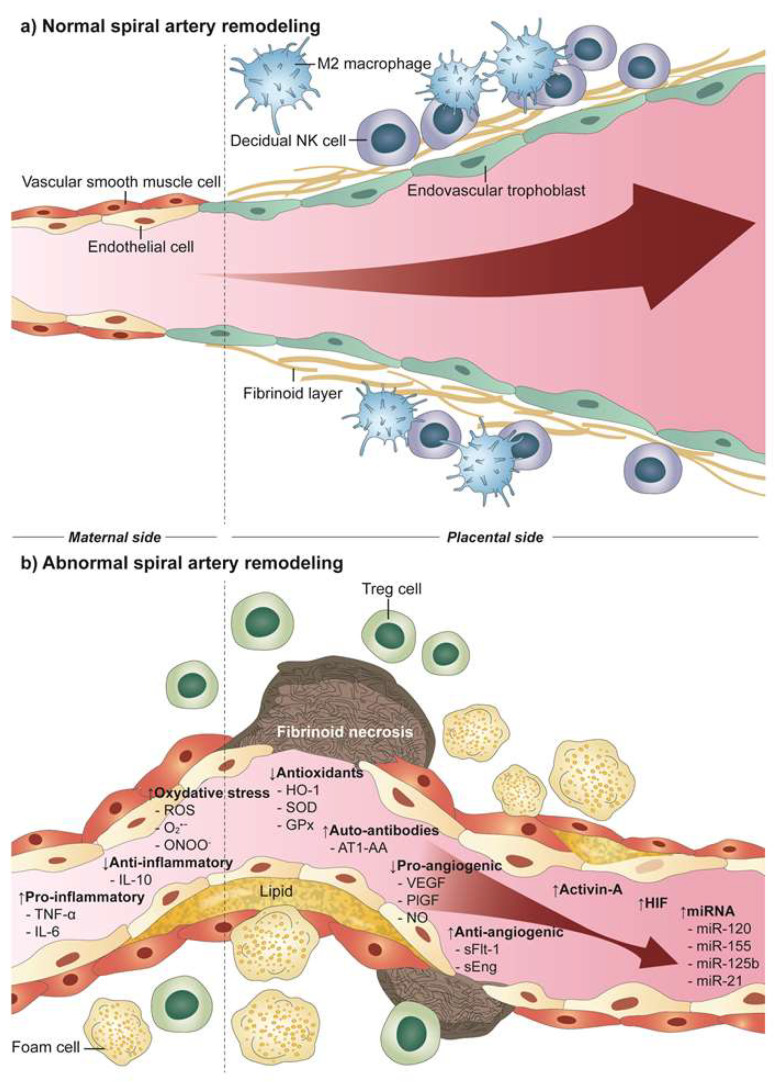
(**a**) Normal placentation with successful spiral artery remodeling. Endovascular trophoblasts become incorporated into the vessel wall and a fibrinoid layer substitutes the original smooth muscle cell layer, resulting in a low resistance vessel with a newly built thin, flexible wall which brings adequate uteroplacental perfusion. (**b**) Abnormal spiral artery remodeling in preeclampsia. Failure of endovascular trophoblast invasion results in a relatively narrow, thick-walled tortuous vessel with high resistance leading to reduced uteroplacental perfusion (RUPP). Atherosis is shown with lipid deposition in walls of spiral arteries with lipid-filled foam cells, perivascular lymphocytic infiltration and vascular fibrinoid necrosis. Various molecular factors resulting from RUPP are listed.

Nevertheless, impaired spiral artery remodeling alone is insufficient to explain the diverse maternal clinical syndromic signs of preeclampsia and how it actually increases the risk of cardiovascular disease after the pregnancy. In women with preeclampsia, intravascular inflammation and dysfunctional lipid metabolism is commonly found, characterized by increased low-density lipoprotein and triglycerides, and decreased high-density lipoprotein [18,19]; these factors lead to lipid deposition in walls of spiral arteries which resemble early stages of atherosclerosis and the vascular lesions are named as ‘atherosis’. Acute atherosis is characterized by the presence of subendothelial lipid-filled foam cells, perivascular lymphocytic infiltration and vascular fibrinoid necrosis, which result from inflammatory stress and arterial damage (Figure 2) [20]. These evidence of vascular inflammation are similarly found in transplant vasculopathy and atherosclerosis in patients with ischemic heart disease [21]. Once present in pregnancy, atherosis lesions can lead to placental dysfunction by narrowing the lumen of spiral arteries which leads to inadequate uteroplacental perfusion [22]. In fact, the prevalence of atherosis is higher in preeclampsia compared to normotensive pregnancies which has been reported as 10–52% and 0.4–11%, respectively [23,24,25,26]. Therefore, atherosis in the maternal vascular system serves as a major risk factor for both early and late onset preeclampsia which eventually leads to stage 1 in the previously mentioned 2-stage model. Moreover, several molecular factors involved in placental dysfunction can also cause acute atherosis, which will be discussed in greater detail below [27]. 

## 6. Molecular Factors Resulting from Inadequate Uteroplacental Perfusion Leading to Preeclampsia

### 6.1. Inflammatory Factors

Placental ischemia due to reduced uteroplacental perfusion pressure (RUPP) increases the release of proinflammatory cytokines. TNF-α is increased in plasma of women with preeclampsia as compared to normal pregnant women [28], which increases vascular permeability and lymphocyte activation and disrupts mitochondrial function leading to oxidative stress [29]. 

Interleukin-6 (IL-6) is elevated in patients with preeclampsia compared to women with normal pregnancy [28]. IL-6 dislocates the tight junctions in endothelial cells which leads to increased vascular permeability and endothelial dysfunction [30]. This has been confirmed in rats with reduced uteroplacental perfusion which showed increased plasma levels of IL-6 with high CD4+ T cell production of inflammatory cytokines [31]. Also, chronic infusion of IL-6 in pregnant rats caused hypertension and proteinuria along with reduced vascular relaxation [32]. 

Interleukin-10 (IL-10) is an anti-inflammatory cytokine which is reduced in the placenta of rats with reduced uteroplacental perfusion and in serum of women with preeclampsia [33,34]. A recent meta-analysis of 56 studies on the circulating IL-10 levels in preeclamptic women revealed that the serum IL-10 levels were not significantly different before the onset of preeclampsia; however, once the clinical syndrome of preeclampsia occurs, IL-10 levels were significantly lower in preeclamptic women compared to normotensive controls (standardized mean differences, −0.79 [95% CI, −1.22 to −0.35]; *p* = 0.0004). Moreover, the decreased level of IL-10 was present in all forms of preeclampsia regardless of its onset and severity [35]. This suggests that IL-10 levels may not be a suitable marker for early detection of preeclampsia, but increasing IL-10 may be a potential therapeutic target of preeclampsia, which could lead to future studies. 

### 6.2. Reactive Oxygen Species (ROS) 

Reactive Oxygen Species (ROS) such as superoxide, hydrogen peroxide, and the hydroxyl ion contains highly reactive oxygen. Pregnancy itself is a state of oxidative stress resulting from placental metabolism and increased maternal metabolic activity, which is counterbalanced by abundant antioxidants [36]. In preeclampsia, decreased expression of antioxidants such as heme oxygenase-1 (HO-1), HO-2, copper/zinc superoxide dismutase (SOD), glutathione peroxidase (GPx) and catalase fails to counterbalance the increased ROS production, leading to lipid peroxidation, increased thromboxane A2 and loss of GPx activity in the placenta [37]. The impaired blood flow in the spiral arteries due to RUPP also mediates an ischemia/hypoxia-reperfusion injury, leading to oxidative changes in placental proteins and lipids, mitochondrial injury, and increased ROS production [38]. In women with preeclampsia, decreased serum levels of the antioxidant ascorbate were shown to be associated with decreased brachial artery flow-mediated dilation, and administration of ascorbic acid improved flow mediated dilation, supporting an association between endothelial dysfunction and oxidative stress in preeclampsia [39].

Moreover, oxidative stress results in reduced bioavailability of nitric oxide (NO), a major vasodilator which regulates blood pressure in placenta [40]. Oxidative stress inhibits nitric oxide synthase (eNOS) which is required for biosynthesis of NO, and the radical anion superoxide (O_2_^•−^) reacts with NO to form peroxynitrite (ONOO^–^), which is a strong pro-inflammatory factor [41].

### 6.3. Angiotensin II (AngII) and Angiotensin II Type 1 Receptor (AT1R) Autoantibodies (AT1-AA)

Angiotensin II (AngII) is an important regulator of blood pressure and electrolyte homeostasis. About 40% of AngII is produced locally in the placenta by chymase, a chymotrypsin-like serine protease, which is a non-angiotensin converting enzyme found mainly in the syncytiotrophoblast of the placenta. AngII via the AngII type 1 receptor (AT1R) promotes vasoconstriction, vascular growth, and inflammation, and increases intracellular free Ca^2+^ concentration and Rho/Rho-kinase activity in vascular smooth muscle. AngII via the endothelial angiotensin II type 2 receptor (AT2R) activates eNOS, and increases production of NO and prostacyclin (PGI2) which oppose AngII-induced vasoconstriction. Although increased plasma levels of renin and AngII is observed in normal pregnancy, the response to AngII is decreased due to decreased expression of AT1R, possibly by AT2R. However, hypoxia in RUPP has been shown to increase the AT1R expression and plasma levels of AngII in rabbits, as well as in human preeclamptic placentas [42,43].

In preeclampsia, AT1R forms a heterodimer with the bradykinin B2 receptor (B2R) called AT1R-B2R protein complex and becomes hyper-responsive to AngII; AT1R-B2R formation is increased in preeclampsia since down-regulation of the protein complex expression is inhibited due to beta-arrestin1 (ARRB1) dysfunction [44]. Therefore, AT1R-B2R has become an emerging treatment target of preeclampsia. The beta-arrestin-biased AT1R agonist, TRV027, is expected to stimulate the AT1R-B2R downregulation—which is impaired in preeclampsia—and recent experiments have shown that it actually lowered blood pressure and prevented symptoms of preeclampsia in animal models [44,45].

AT1-AA are agonistic autoantibodies to the AT1R that mediates vascular signaling via protein-1, calcineurin, and nuclear factor kappa B (NFκB). AT1-AA induces the secretion of plasminogen activator inhibitor-1 (PAI-1) which inhibits trophoblast invasion, increases ROS, increases intracellular free Ca^2+^ concentration, activates the tissue factor causing thrombosis, and increases blood pressure [46]. Moreover, AT1-AA along with circulating cytokines stimulate endothelial cells to produce endothelin-1 (ET-1) in preeclampsia, which is a major endothelium-derived vasoconstrictor [47]. Infusion of CD4+ T cells obtained from preeclamptic women in pregnant rats stimulates the immunoglobulin release from B-cells which in turn increases AT1-AA production while inhibition of B-cells reduces AT1-AA mediated hypertension in these rats [48]. Therefore, AT1-AA serves as a possible therapeutic target for treating preeclampsia. Moreover, previous studies have shown that maternal AT1-AA persisted up to 27 months after pregnancy in 17.2% of women with preeclampsia compared to 2.9% in women with normotensive pregnancy [49]. Recently, a follow up study on circulating AT1-AA levels at five to eight years postpartum was published which showed that AT1-AA was persistently found in women with a history of preeclampsia, which might relate to their future CVD risk [50].

### 6.4. Angiogenic/Antiangiogenic Factors

Angiogenic factors are most highly expressed in early pregnancy and are responsible for placental angiogenesis and increasing placental mass that follows fetal growth [51]. Previous studies have revealed that RUPP leads to altered concentrations of pro- and anti-angiogenic factors in women with preeclampsia, which leads to endothelial dysfunction and suggests that they are responsible for the pathology of maternal clinical manifestations of preeclampsia [52]. 

#### 6.4.1. Vascular Endothelial Growth Factors (VEGF)

The VEGF family includes [VEGF-A, VEGF-B, VEGF-C, VEGF-D and placental growth factor (PlGF)], and their receptors [VEGFR-1/fms-like tyrosine kinase-1 (Flt-1), VEGFR-2/kinase insert domain receptor (KDR), VEGFR-3/fms-like tyrosine kinase receptor-4(Flt-4)]. Vascular endothelial growth factor (VEGF) is highly expressed in decidual cells and invading cytotrophoblasts in normal pregnancy, which leads to endothelial cell proliferation for newly developing capillaries in uteroplacental circulation [53]. Moreover, VEGF-A regulates trophoblast functions such as proliferation, differentiation, and invasion, mainly through the Flt-1 and KDR receptors [54]. In preeclampsia, the circulating level of VEGF is decreased and this has been confirmed in studies with RUPP-induced rats in which the VEGF level is also reduced [55,56].

#### 6.4.2. Placental Growth Factor (PlGF)

Placental growth factor (PlGF), a member of the VEGF family, is another proangiogenic factor that binds to Flt-1 which augments the angiogenic effect of VEGF. PlGF exerts not only direct effects on endothelial cells, but also indirect effects on nonvascular cells with pro-angiogenic activity by altering the functioning of immune cells; it recruits monocytes and activates macrophages which can release angiogenic factors, and encourages proliferation of mesenchymal fibroblasts and attracts myeloid progenitors to develop sprouts and collateral vessels [57]. Moreover, PlGF promotes vasodilation of uteroplacental circulation [36]. However, the circulating level of PlGF is decreased in preeclampsia compared to normal pregnancy, which leads to increased vascular resistance in preeclampsia [58]. Therefore, the National Institute for Health and Care Excellence guideline has recommended that obstetricians to utilize maternal serum PlGF levels to rule out preeclampsia in pregnant women with chronic hypertension or who are at a high risk of developing preeclampsia [59].

#### 6.4.3. Soluble FMS-Like Tyrosine Kinase I (sFlt-1)

As a VEGF receptor, Flt-1 is highly expressed in the invading extravillous trophoblasts in the first trimester, which implies that VEGF-Flt-1 interactions lead to early trophoblast invasion [60]. As gestational age develops, VEGF-Flt-1 interaction also guides trophoblast differentiation and migration [61]. Soluble FMS-like tyrosine kinase I (sFlt-1) is a truncated protein resulting from splicing of Flt-1 which lacks the cytoplasmic and transmembrane domain but keeps the ligand-binding domain [62]. Therefore, sFlt-1 antagonizes and inhibits VEGF and PlGF by binding to them and blocking their interaction with Flt-1 for proangiogenic function. In preeclampsia, placental ischemia resulting from RUPP may stimulate upregulation of sFlt-1 by binding of hypoxia inducible factor (HIF) to the promotor of Flt-1 gene [55]. The elevated maternal serum level of sFlt-1 in preeclampsia has been found to be associated with severe endothelial dysfunction and inhibition of VEGF and PlGF by sFlt-1 serves a major pathogenic role in hypertension and proteinuria [5]. VEGF is responsible for decreasing vascular tone and blood pressure by inducing nitric oxide and prostacyclins that have a vasodilatory effect in endothelial cells, which is blocked by sFlt-1. In addition, several molecular mechanisms of sFlt-1 found to be responsible for renal dysfunction are related to glomerular capillary endotheliosis, dysregulation of the glomerular filtration apparatus, and podocyte loss [63]. Therefore, excess of sFlt-1 results in the characteristic antiangiogenic state of preeclampsia which manifests as the clinical syndrome of endothelial dysfunction. In fact, maternal serum level of sFlt-1 to PlGF ratio (sFlt-1/PlGF ratio) can be used as a reliable biomarker for predicting development and severity of preeclampsia [64]. Moreover, a recent systematic review and meta-analysis on the performance of the sFlt-1/PlGF ratio in predicting adverse outcomes in women diagnosed or suspected of preeclampsia showed that the sFlt-1/PlGF ratio performs better in predicting women with early onset preeclampsia in comparison to those with late onset [65]; this relates to our previous topic in chapter 4 which described that defective uteroplacental vascular remodeling is mostly seen in the early onset type of preeclampsia.

#### 6.4.4. Soluble Endoglin (sEng)

Soluble endoglin (sEng), a coreceptor for transforming growth factor-β1 (TGF-β1), is another antiangiogenic factor released by the placenta that acts in synergy with sFlt-1. Endoglin (Eng) is an angiogenic receptor expressed mainly on the surface of placental syncytiotrophoblast and endothelial cells which serves as a co-receptor of angiogenic TGF-β signaling [66]. TGF-β is known to contribute to angiogenesis and appropriate vascular relaxation by increasing VEGF [67,68]. However, in preeclampsia sEng is released in excessive quantity and binds to free TGF-β1 which inhibits the pro-angiogenic TGF-β1 signaling in the vasculature. The circulating level of sEng is elevated in patients with preeclampsia two-to-three months prior to the onset of clinical symptoms and its serum levels seem to be correlated with the severity of the disease [69]. 

### 6.5. Activin A

Activin A is a dimeric glycoprotein belonging to the TGF-β family produced by the placenta and fetal membranes [70]. In preeclampsia, the serum level of activin A is elevated (up to 10-fold) compared to normal pregnancy and it is found to be resulting from increased placental production triggered by oxidative stress [71,72]. In fact, circulating levels of activin A have shown to rise months prior to the onset of the clinical manifestation of preeclampsia, which is earlier than the elevation of sFlt-1 or sEng [73]. Recent studies have shown that elevated activin A in preeclampsia may be responsible for the endothelial dysfunction, which was shown as hypertension, proteiunuria, fetal growth restriction, and preterm littering in activin administered mice [74]. An in vitro study using human umbilical vein endothelial cells (HUVECs) has suggested that activin A up-regulates transcription of endothelial vasoconstrictors such as ET-1 [75]. Moreover, an elevated activin A level had been reported to be strongly correlated with myocardial dysfunction at 1 year after preeclamptic pregnancy, and a recent follow up study confirmed that the activin A level still remained elevated with impaired cardiac function 10 years after preeclamptic pregnancy, implying its potential use as a tool for monitoring women at risk for postpartum CVD [76,77].

### 6.6. Hypoxia Inducible Factor 

Hypoxia inducible factor (HIF) is a heterodimer consisting of HIF1-α and HIF2-α subunits, which are regulated by oxygen, and a constitutively expressed HIF1-β subunit. In a hypoxic environment, HIF-1 regulates transcription of various genes, including VEGF, TGF-β3, and NOS, by binding at their promotor and enhancer regions [36]. HIF expression is shown to be higher in normal pregnancy, probably due to high estrogen and progesterone levels; however, HIF-1α and HIF-2α is overexpressed further in preeclampsia in response to RUPP [78,79]. Moreover, HIF-1α upregulates anti-angiogenic factors such as sFlt-1, sEng, and ET-1 expressions and AngII and AngII-converting enzyme (ACE) expressions in the lungs and kidney which add on to the abnormal placentation and development of preeclampsia [80]. An animal study with RUPP rats showed that inhibition of HIF-1α using siRNA reversed the high blood pressure, renal damage, proteinuria, and elevated serum sFlt-1 level [81]. Therefore, the efficacy of using maternal serum level of HIF-1α as a predictive marker for preeclampsia has been questioned. A recent prospective study showed that high serum HIF-1α level (above 1.45 MoM) in the first trimester of pregnancy (11–13^+6^ weeks of gestation) was related to development of preeclampsia, which requires further confirmation with large-scaled studies [82].

### 6.7. MicroRNAs

MicroRNAs(miRNAs) are small (<25 nucleotides), single-stranded, non-coding RNAs that regulate gene expression by inhibiting translation. These molecules bind to the untranslated lesion of a target gene and silence their expression [83]. During pregnancy, miRNAs are profusely expressed in the placenta, mainly from villous trophoblasts, and play pivotal role in several processes including trophoblast proliferation, immune tolerance, and angiogenesis [84]. 

Specifically, miR-210 has been reported to be overexpressed in placentas of preeclampsia [85]. Studies have shown that miR-210 is strongly linked with hypoxia related to RUPP which leads to inadequate trophoblast invasion and failure of spiral artery remodeling in preeclampsia [86]. miR-210 is upregulated by HIF which overexpresses it in response uteroplacental hypoxia in order to regulate genes involved in various pathways including angiogenesis, inflammation, and cell proliferation [87]. Another miRNA involved in preeclampsia is miR-155, which has been shown to inhibit cysteine-rich protein 61 (CYR61), an essential angiogenic factor in pregnancy [88,89]. A crucial function of CYR61 is related to inducing the expression of VEGF, which is a major pro-angiogenic factor as previously mentioned [87]. Previous studies have shown that CYR61 gene expression is downregulated in preeclamptic placentas compared to those of normal pregnancy, and suggested that increased miR-155 causes inhibition of the CYR61-VEGF pathways, which leads to reduced placental angiogenesis [90].

Additionally, miR-125b is known to be an anti-angiogenic factor which decreases VEGF expression when it is overexpressed [91]. A recent case-control study showed that the maternal plasma level of miR-125b at 12 weeks of gestation is significantly elevated compared to those in normal pregnancy. Moreover, the same study revealed that miR-125b targets trophoblast cell surface antigen-2 (Trop-2) protein in placental tissue, suggesting miR-125b might be involved in development of preeclampsia via modulating Trop-2 expression in the syncitiotrophoblast [92].

The role of miR-21 in preeclampsia has been also newly studied, since it regulates the forkhead box M1 protein (FOXM1), which is expressed in cytotrophoblasts for proliferation and differentiation, responsible for the early placental development [93]. In fact, a study showed that miR-21 is elevated with reduced FOXM1 expression in patients with preeclampsia compared to those in normotensive pregnant women, implying that miR-21 may impede the early placental invasion leading to preeclampsia [94]. These results demonstrate that various miRNAs are involved in the pathway of preeclampsia which implies their potential to become possible future therapeutic targets for treatment of preeclampsia. 

## 7. Preeclampsia and Future Cardiovascular Health

As discussed so far, the key mechanism of preeclampsia is disrupted spiral artery remodeling resulting in insufficient blood flow to the placenta, which in turn leads to RUPP. RUPP leads to a change in the level of several factors released by the placenta—mainly an increase of pro-inflammatory and anti-angiogenic factors and a decrease of pro-angiogenic factors—which eventually spread into the maternal circulation (Figure 2); this results in endothelial dysfunction leading to disrupted maternal hemodynamics. Although some aspects of maternal vascular damage return to normal after birth, it has become apparent that the endothelial damage is evidenced to persist beyond the acute disease in pregnancy.

Cardiovascular diseases (CVD) are currently considered as the leading cause of death globally [95]. Women with a history of HPD, including preeclampsia, have a twofold increased risk of future CVD compared with women with normotensive pregnancies [96,97,98,99,100]. HDP, by definition, is development of de novo hypertension after 20 weeks of pregnancy which includes the following 4 categories: (1) preeclampsia/eclampsia; (2) gestational hypertension; (3) chronic hypertension; and (4) preeclampsia/eclampsia variants superimposed on chronic hypertension [101]. The epidemiologic research has reported a significantly rising prevalence of preeclampsia and gestational hypertension (by 25 and 184%, respectively) in the last decade. The worldwide population based increases in well-known risk factors for preeclampsia such as pre-pregnancy overweight and obesity, diabetes, twin pregnancy, and advanced maternal age are expected to contribute to the growing rates of the overall HDP [102]. Although it is controversial whether gestational hypertension and preeclampsia develop from the same pathophysiological mechanisms, they both share similar pathway which involves placental insufficiency leading to systemic endothelial dysfunction [103]. In addition, the rate of progression into preeclampsia ranges from 15% to 46% of women diagnosed with gestational hypertension, suggesting that gestational hypertension and preeclampsia may be considered as different stages of a continuous process with an identical pathophysiology of angiogenic imbalance rather than as separate distinct diseases [103]. 

Recently, a history of HDP has been designated as an independent risk factor for future cardiovascular events and incorporated in guidelines for risk stratification of stroke and CVD [104,105]. However, whether HDP serves to unmask a preexisting high CVD risk in an individual or HDP is causally associated with vascular remodeling leading to the development of postpartum CVD remains unclear. The former hypothesis is supported by the fact that HDP and CVD share the preexisting risk factors in common, such as obesity, hypertension, hyperinsulinemia, hyperlipidemia and a family history [106,107,108,109,110]. Therefore, a woman with an already existing high risk of CVD is revealed by the “stress test” of pregnancy as a clinical manifestation of HDP. On the other hand, the latter theory suggests that pro-atherogenic stress of HDP could activate arterial wall inflammation and induce changes in vasculature which may result in future CVD. However, the two mechanisms are not completely exclusive of each other since they both involve endothelial dysfunction caused from vascular maladaptation during pregnancy which we have discussed previously in chapter 4 [111]. Therefore, a history of HDP increases risk of future CVD in a dose dependent manner which depends on how severely the uteroplacental circulation is compromised. The clinical manifestations of HDP with earlier onset, iatrogenic preterm delivery, fetal growth restriction and recurrence of HDP in subsequent pregnancies are related to greater risk of future CVD [112]. In terms of timing, it has been known that within one or two decades after delivery, women with a history of HDP are more likely to experience premature cardiovascular events, such as symptomatic heart failure, myocardial ischemia, and cerebral vascular disease [112,113]. Two systematic reviews and meta-analyses demonstrated that the increased risk for CVD and hypertension is greater during the first 10 years after a pregnancy affected by HDP compared to the risk past 10 years after the affected pregnancy [97,114]. Up to one third of women with a history of HDP may develop hypertension within a decade of the affected pregnancy before middle age, indicating that they are more likely to develop CVD at a much younger age compared to controls [115]. Despite the previous belief that the physiological changes and the resulting cardiovascular stress during pregnancy return to pre-pregnancy levels shortly after delivery, the endothelial damage in HDP and its related factors seem to persist and exert long-term consequences on maternal cardiovascular health [116].

### 7.1. Chronic Hypertension

The nationwide register-based cohort study of 1.5 million women who had delivered in Denmark from 1978 to 2012 has reported that women with gestational hypertension had the highest risk of developing chronic hypertension after pregnancy, followed by women with severe preeclampsia and moderate preeclampsia. In fact, of women in their 20 s, 14% of nulliparous women with HDP developed hypertension in the first 10 years after pregnancy compared with 4% of nulliparous women with normotensive pregnancy. The corresponding percentages of developing hypertension after HDP and without HDP in their forties were higher as expected: 32% and 11%, respectively. In fact, in a year after delivery, women with HDP had 12-fold to 25-fold higher rates of hypertension compared to women with normotensive pregnancy—which remained high as 3 to 10-fold in the first 10 years after pregnancy and twice higher when it passes 20 years postpartum. The decreasing trend in the relative risk of developing chronic hypertension as time passes from delivery (compared to controls) seem to be due to the natural behavior of hypertension in which its baseline risk increases with age. Therefore, it can be concluded that the risk of hypertension associated with HDP is highest shortly after an affected pregnancy and persists for more than 20 years [115]. The PROSPECT cohort consisting The European Prospective Investigation into Cancer and Nutrition (EPIC)-NL cohort demonstrated that women with HDP reported a diagnosis of hypertension 7.7 years earlier (95% confidence interval [CI] 6.9–8.5) than women with a normotensive pregnancy, and that women with HDP have an increased risk of developing hypertension (odds ratio 2.12, 95% CI; 1.98–2.28) [117].

The American Heart Association has reported the Heart Disease and Stroke Statistics—2021 Update with an enhanced focus on adverse pregnancy outcomes (APO). APOs include HDP, gestational diabetes, preterm delivery, and small for gestational age. These interrelated disorders are associated with long-term risk of cardiometabolic disease in maternal and offspring [118]. While normal pregnancy without APO can be recognized as proper maternal cardiometabolic adaptation, APOs may reflect a maladaptive response to the “stress test” of pregnancy. According to the prospective observational cohort study named Nulliparous Pregnancy Outcomes Study Monitoring Mothers-to-be Heart Health Study, the incidence of APO was 22.7%, as 1017 out of 4484 females were affected (22.7%). In this study, the overall incidence of hypertension was 5.4% (95% CI; 4.7–6.1%), with an increased risk among females with any APO (defined as HDP, small-for-gestational-age birth, preterm delivery, and stillbirth) and by subtype (any HDP: Relative risk (RR), 2.7 [95% CI: 2.0–3.6]; preeclampsia: RR, 2.8 [95% CI: 2.0–4.0]; preterm delivery; RR, 2.7 [95% CI: 1.9–3.8]). Among APO, women with both HDP and iatrogenic preterm delivery had the highest risk of hypertension (RR 4.3, 95% CI: 2.7–6.7) in a short-term follow-up over a mean of 3.2 years after the first pregnancy. Under such conditions, chronic hypertension developed in 45.4% of females [119]. A recurrence rate of developing preeclampsia in the subsequent pregnancy has been reported as 16% [120]. Recurrent preeclampsia is consistently associated with a higher risk ratio of developing chronic hypertension (RR 2.3, 95% CI; 1.9–2.9) than women with a subsequent uncomplicated pregnancy after a preeclamptic pregnancy [121]. Other longitudinal prospective studies demonstrated that women with a history of preterm preeclampsia had a higher prevalence of hypertension compared with term preeclampsia [122]. Overall, it has been postulated that HDP affects the future risk of developing chronic hypertension in a dose-dependent manner as mentioned previously, depending on the severity of placental insufficiency which manifests clinically; HDP with earlier onset, severe criteria, preterm delivery, fetal growth restriction or recurrence affects the risk of postpartum hypertension with a greater chance [112].

### 7.2. Altered Vascular Structure 

Carotid intima-media thickness (CIMT) is a well validated, non-invasive marker of subclinical atherosclerotic disease. It involves ultrasound evaluation of the thickness of the intimal and medial carotid arterial wall. Data have shown that an increased CIMT confers an elevated risk of coronary atherosclerotic lesions and future cardiovascular disease in both histological and epidemiological studies [123]. Although an assessment of CIMT could reflect arterial remodeling in HDP such as changes in arterial internal diameter and wall thickness, there are only few small-scale studies demonstrating heterogenous results of CIMT assessment in pregnancy. 

A prospective study of women with late onset preeclampsia reported a significant increase of 108μm in CIMT compared with those in normotensive pregnancy (459 ± 95 vs. 351 ± 85 μm, *p* = 0.0001) [124]. While early onset preeclampsia was also characterized by increased CIMT in another study, it reported that mean CIMT did not differ significantly between the early onset preeclampsia group and the normotensive pregnancy group, but it was significantly increased in the late onset preeclampsia group as compared to controls [125]. On the other hand, another prospective cohort study has identified increased CIMT in only pregnant women with underlying chronic hypertension rather than in women with preeclampsia [126]. Despite the heterogenous study results and inconclusiveness, these studies suggest the possibility that vascular structural changes may reflect vascular remodeling as adaptive response to hypertension during pregnancy.

The coronary artery calcification (CAC) Score assessed by coronary computed tomography (CCT) is another important predictor for CVD events. Calcification is related to arterial stiffness associated with an increased risk of CVD, and the St. Francis Heart study determined that for a CAC score > 100 Agaston units (AU), the relative risk for atherosclerotic CVD was 9.6 (95% CI; 6.1–13.9) [123]. Significant coronary atherosclerosis could be identified by imaging the non-calcified coronary plaque with contrast-enhanced coronary CT angiography (CCTA) before calcification of plaque occurs [127]. Although there has been no study for evaluation of CAC score during pregnancy due to the fetal risks of radiation exposure, several studies have reported that CAC score is higher in postpartum women with a history of HDP. In the first prospective cohort study, a history of preeclampsia was shown to be associated with an increased risk of CAC > 30 years after affected pregnancies, even after controlling individually for traditional risk factors. Compared to women with uncomplicated pregnancies, it demonstrated that the odds of having a higher CAC score was 3.54 (CI; 1.39–9.02) times greater in women with prior preeclampsia without adjustment and 2.61 (CI; 0.95–7.14) times greater after adjustment for current hypertension [128]. Regarding asymptomatic women aged 45 to 55 years, it has been also reported that 30% of women with a history of preeclampsia show features of coronary atherosclerosis on vascular computed tomography imaging as compared to 18% of women from the reference group [129]. In addition, a recent study showed that women with previous preeclampsia developed CAC about five years earlier than women with normotensive pregnancies [130]. Population based studies also showed that women with a history of preeclampsia develop cardiovascular risk factors, including high blood pressure, dyslipidemia, and diabetes, five to ten years earlier than women without such a history [130]. Therefore, early cardiovascular screening of women with a history of HDP would definitely benefit in reducing future CAD events.

### 7.3. Coronary Artery Disease and Cerebrovascular Accident

Atheromatous plaque is formed through a chronic, inflammatory progression of atherosclerotic disease without overt symptoms. It can result in a sudden occurrence of coronary artery disease (CAD), such as myocardial infarction, unstable angina pectoris and cardiac death [131]. The prevalence of ischemic heart disease in women with a history of HDP is significantly higher than compared to those with a history of normotensive pregnancy [4,132]. As it was seen in chronic hypertension discussed previously, HDP also affects future CAD risk depending on its severity, association with APO, and recurrence; these associations remained significant after adjustment for confounding variables [121,133]. A previous meta-analysis published in 2017 reported that the future risk of CAD following HDP was not significant after adjustment for pregestational hypertension [95]. However, nine large cohort studies from various countries including Norway, the United Kingdom, Denmark, United States, Canada, and Australia were followed, and a recent systematic review and meta-analysis of 73 studies involving > 13 million women demonstrated that the overall combined relative risks of CAD for women with a history of HDP compared with the normotensive group was 1.66 (CI;1.49–1.84), along with 1.80 (CI; 1.67–1.94) for any CVD, 2.87 (CI; 2.14–3.85) for heart failure, 1.60 (CI; 1.29–2.00) for peripheral vascular disease, 1.72 (CI;1.50–1.97) for stroke, 1.78 (CI; 1.58–2.00) for CVD-related mortality, and 3.16 (CI; 2.74–3.64) for chronic hypertension [114,133,134,135,136,137,138].

In addition, the risk ratio of cerebrovascular disease (CD) in women with previous preeclampsia ranged from 1.53 to 3.13 [95,117,139,140]. Regarding women with previous gestational hypertension, a prospective population-based cohort study reported that women with a gestational hypertension had a 1.3-fold (CI; 0.9 to 1.7) higher risk of developing CD compared with women without any HDP [141]. Furthermore, a meta-analysis in 2018 found that recurrent preeclampsia was consistently related to an increased risk ratio of stroke (RR 1.7, CI; 1.2–2.6) when compared with preeclampsia in a single pregnancy followed by subsequent uncomplicated pregnancies [121]. 

### 7.4. Postpartum Management after HDP

Although the mechanisms still remain elusive, it is evident that the risk of CVD rises after HDP, which can occur early in the postpartum period. As of today, various guidelines for postpartum follow-up are suggested. 

The American College of Obstetricians and Gynecologists (ACOG) recommends that all women with HDP have an initial check with their obstetrician within three weeks postpartum, followed by another visit at three months postpartum, and annual cardiovascular checkups consequently [142]. A recent recommendation by the American Heart Association suggests initiation of screening for CVD as soon as possible after delivery in women with HDP. The suggested strategies include: (1) an interdisciplinary approach for early identification of CVD risk factors, (2) continuous postpartum visits starting six to eight weeks postpartum and annually thereafter, (3) consistent monitoring of blood pressure and biomarkers at clinic and at home, and (4) educating patients about their individual risk and helpful lifestyle modifications to prevent CVD [143]. 

## 8. Conclusions

The key mechanism of preeclampsia is defective spiral artery remodeling resulting in inadequate blood flow to the placenta, which in turn leads to RUPP. RUPP leads to rise in pro-inflammatory and anti-angiogenic factors and decrease in pro-angiogenic factor in maternal circulation which in turn results in endothelial dysfunction leading to disrupted maternal hemodynamics. The association between HDP and postpartum CVD has become a well-accepted fact, especially among women with a history of preeclampsia. Risk factors of CVD mostly develop in the early postpartum period and the most vulnerable timing for CVD events is the decade after delivery, in which the related circulating factors persist in maternal circulation. Therefore, women with a history of HDP should be considered for risk evaluation before the current cardiovascular screening guidelines. Although the exact linking mechanisms between HDP and CVD are still left for further research, primary prevention strategies to reduce cardiovascular disease in women should always include risk stratification based on their obstetrical history.
